# A briefly overview of the research progress for the abscisic acid analogues

**DOI:** 10.3389/fchem.2022.967404

**Published:** 2022-07-22

**Authors:** Yaming Liu, Shunhong Chen, Panpan Wei, Shengxin Guo, Jian Wu

**Affiliations:** State Key Laboratory Breeding Base of Green Pesticide and Agricultural Bioengineering, Key Laboratory of Green Pesticide and Agricultural Bioengineering, Ministry of Education, Center for R&D of Fine Chemicals of Guizhou University, Guiyang, China

**Keywords:** abscisic acid (ABA), analogues, biological activity, mechanism of action, research progress

## Abstract

Abscisic acid (ABA) is an important plant endogenous hormone that participates in the regulation of various physiological processes in plants, including the occurrence and development of somatic embryos, seeddevelopment and dormancy. ABA is called “plant stress resistance factor”, while with the limitation of the rapid metabolic inactivation and photoisomerization inactivation of ABA for its large-scale use. Understanding the function and role of ABA in plants is of great significance to promote its application. For decades, scientists have conducted in-depth research on its mechanism of action and signaling pathways, a series of progress were achieved, and hundreds of ABA analogues (similar in structure or function) have been synthesized to develop highly active plant growth regulators and tools to elucidate ABA perception. In this review, we summarize a variety of ABA analogues, especially the ABA receptor analogues, and explore the mechanisms of ABA action and catabolism, which will facilitate the development of novel ABA analogues with high biological activities.

## Introduction

The environment that plants depend on for survival is constantly changing, and some adverse conditions, such as extreme temperature, drought, salinization and other abiotic stresses, can seriously affect the growth and development of plants (Young, 2007). Future climate change will lead to a gradual increase in the frequency (Guo et al., 2021). Therefore, it is important to improve the adaptability of plants to these abiotic stresses, especially drought.

Plant hormones play an important role in regulating plant adaptation to abiotic stresses (Waadt et al., 2022). Abscisic acid (ABA) synthesis is one of the fastest responses to stresses in plants (Zhao et al., 2018). ABA is a phytohormone which is named for its function in inhibiting plant growth and promoting leaf shedding. Subsequently, more and more studies show that ABA is also involved in regulating other growth and development processes of plants, such as seed germination, dormancy and stomatal closure. Moreover, ABA has been reported to control the expression of many stress-responsive genes and involve in many kinds of stress responses in plants (Hoth et al., 2002; Seki et al., 2002; Nemhauser et al., 2006).

The studies on the function and application for ABA have made remarkable achievements in the past 20 years. The synthesis, perception and transduction of ABA signals have been summarized (Young, 2007; Fidler et al., 2015; Zhang, et al., 2015), Asghar, Ma and co-workers had surveyed the advances from the functions of ABA (such as the effects on osmotic stress or on the seed dormancy, germination, and plant resistance to transpiration), respectively (Ma et al., 2018; Asghar et al., 2019). However, few summaries for ABA derivatives and structural analogues (especially studies after 2014), biosynthesis, transport and catabolism have not been made. Therefore, this article summarizes the research overview for the ABA based on the following aspects: Firstly, the discovery of ABA receptor structure and signal transduction mechanism, as well as the regulation of ABA on plants under abiotic stress is introduced. Secondly, the current discovery of ABA analogues and their functions are summarized. Finally, the existing challenges are discussed. This brief article gives an overview on the progress for the ABA analogues in the past decades and introduces the latest progress. The aim for this briefly article provides readers with a convenient route to touch this topic, and hopefully serve some educational purpose for graduate students and assist further research in related fields.

## ABA biosynthesis, transport and catabolism

**FIGURE 1:**
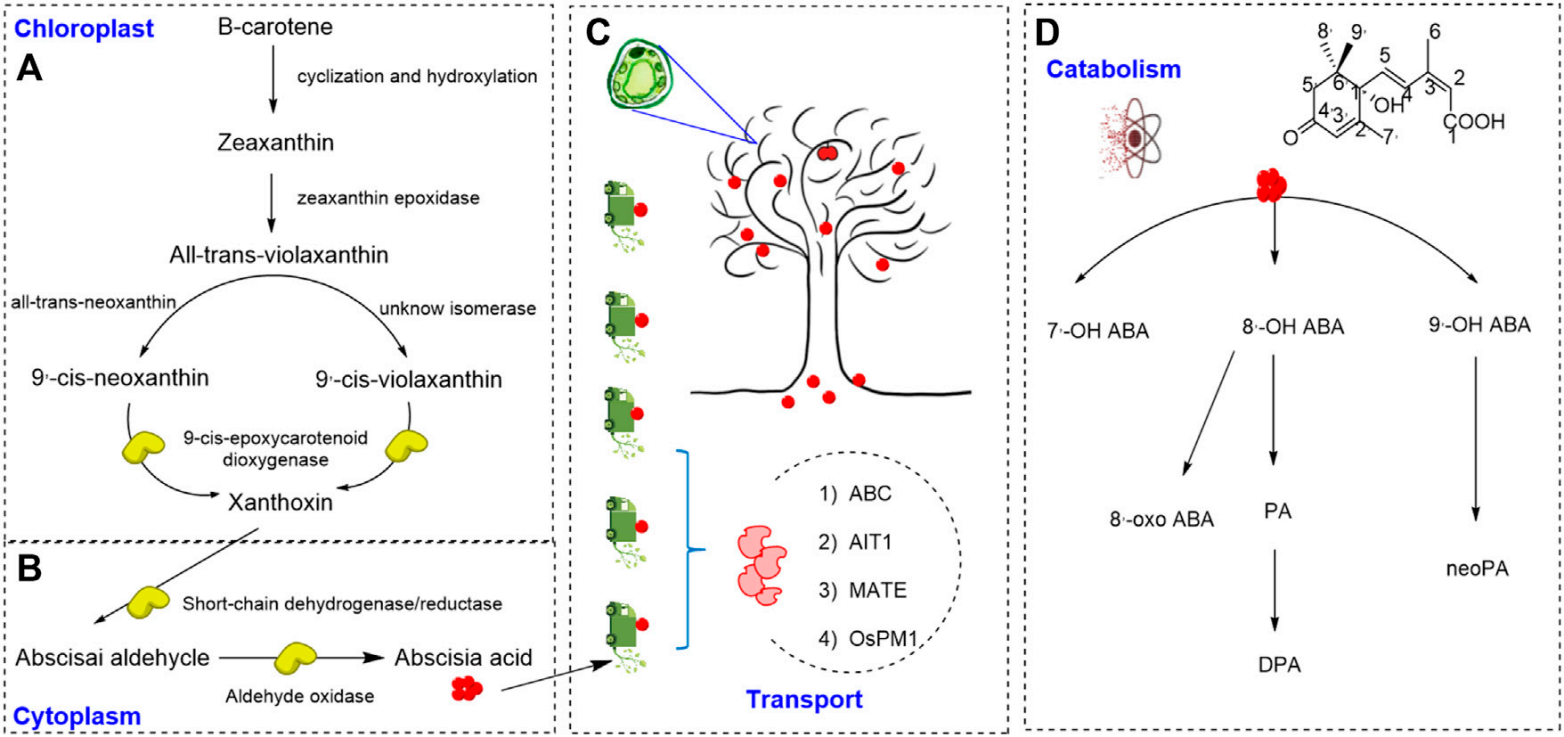
ABA biosynthesis, transport and catabolism **(A,B)**. Biosynthetic pathway of abscisic acid (Izquierdo-Bueno et al., 2018; Takino et al., 2018). **(C)** Transport form of abscisic acid (Kuromori et al., 2010; Kang et al., 2015). **(D)** The catabolic pathway of abscisic acid (Nambara and Marion-Poll, 2005).

As shown in Figures 1A,B, ABA is a sesquiterpenoid phytohormone containing 15 carbon atoms. ABA is synthesized in plants mainly using the C_40_ indirect pathway (also known as the carotenoid pathway), which is initiated from the cleavage of a C_40_ precursor known as β-carotene (Izquierdo-Bueno et al., 2018.; Takino el al., 2018). After cyclization and hydroxylation, β-carotene is converted to zeaxanthin (C_40_), which is catalyzed by zeaxanthin epoxidase (ZEP) to form all-trans-violaxanthin (C_40_). This pathway then bifurcates into two pathways. All-trans-violaxanthin can be further converted to 9′-cis-neoxanthin (C_40_) through all-trans-neoxanthin (C_40_), or it is converted to 9′-cis-violaxanthin (C_40_) directly by an unknown isomerase (North et al., 2007). Then, 9′-cis-violaxanthin and 9′-cis-neoxanthin both can be catalyzed by 9-cis-epoxycarotenoid dioxygenase (NCED) to produce xanthoxin (the C_15_ precursor of ABA), which can also act as a growth inhibitor (Anstis et al., 1975; Schwartz et al., 1997). Xanthoxin is presumed to migrate from the plastid to the cytosol (Nambara and Marion-Poll, 2005), where it is converted to abscisic aldehyde. Then abscisic aldehyde is eventually oxidized to ABA by abscisic aldehyde oxidase (AAO3) (Bittner et al., 2001; Cheng et al., 2002).

Recent studies showed that stress-induced ABA biosynthesis primarily occurs in vascular tissues and leaves, but ABA functions in various cells (Kuromori et al., 2010; Zhang et al., 2018). Therefore, it is important for ABA to transport among different organs, especially in the systemic stress responses of the whole plant. The active transport of ABA is mediated by many factors, such as ATP-binding cassette (ABC) transporters (Kuromori et al., 2010; Kang et al., 2015), ABA-IMPORTING TRANSPORTER1 (AIT1, which is also known as low-affinity nitrate transporter NRT1.2) (Kanno et al., 2012), DTX type/multidrug and toxic compound extrusion (MATE) transporters (Yu et al., 2014), and AWPM 19 family proteins (OsPM1) (Yao et al., 2018). These ABA transporters have been increasingly shown to be involved in stress responses, which are shown in the Figure 1C.

The catabolism of ABA in plant is controlled via two pathways: hydroxylation and conjugation, as shown in Figure 1D (Nambara and Marion-Poll, 2005). ABA can be hydroxylated via oxidation at three positions of the ring structure, C-7′, C-8′, and C-9′, triggering further inactivation steps, of which C-8′ is the primary site in plants (Cutler and Krochko, 1994). The unstable 8′-hydroxy ABA is converted to phaseic acid (PA) (Kushiro et al., 2004; Nambara and Marion-Poll, 2005), and PA has been reported to selectively activate a subset of ABA receptor PYLs (Weng et al., 2016). PA is then converted to dihydrophaseic acid (DPA), which is further catalyzed to DPA-4-O-β-D-glucoside (DPAG) (Weng et al., 2016). ABA conjugation is another pathway to regulate cellular ABA amounts under both normal and dehydration conditions (Lee et al., 2006; Xu et al., 2012). ABA and hydroxy ABA are conjugated with glucose for inactivation, forming different conjugates. The ABA-glucose ester (ABA-GE) is the predominant form of these conjugates and stored in vacuoles and the apoplast (Dietz et al., 2000; Liu et al., 2015). When the environment changes such as dehydration, ABA-GE is rapidly transformed to active ABA by β-glucosidases (Lee et al., 2006; Xu et al., 2012). The conjugation/deconjug-ation cycle enables plants to phenotypically adapt to their environment through ABA-mediated responses by activating and inactivating ABA rapidly (Chen et al., 2020).

## ABA signal transduction and the role of ABA pathways in stress signaling

The ABA signaling pathway has been studied for several decades, and the core ABA signaling components including three protein classes: ABA receptors, negative regulators and positive regulators (Danquah et al., 2014). ABA functions in plants through cellular recognition by the intracellular receptor. Many ABA receptors have been reported so far, such as H subunit of Chloroplast Mg^2+^-chelatase, G-protein coupled Receptor 2, GPCR-type G protein, and Pyracbactin Resistance/Pyracbactin resistance-like/Regulatory Component of ABA Receptor (PYR/PYL/RCAR) (Guo et al., 2011).

PYR/PYL/RCARs are the most important among these receptors, the discovery of which basically elucidates the ABA signaling pathway (Ma et al., 2009; Park et al., 2009). Most plants encode more than one PYR/PYL/RCAR proteins, and they are differentially expressed in multiple organs, cells during different growth stages (Antoni et al., 2013; Dittrich et al., 2018). Genetic evidences showed that a high level of functional redundancy exists in this gene family, but they are essential for ABA perception, signal transduction and response to stress in plants. PYR/PYL/RCAR mutants displayed impaired ABA responses (Miao et al., 2018; Zhao et al., 2018), while overexpression of PYR/PYL/RCARs enhanced ABA responses to impact abiotic stress tolerance (Santiago et al., 2009; Mega et al., 2019).

**FIGURE 2:**
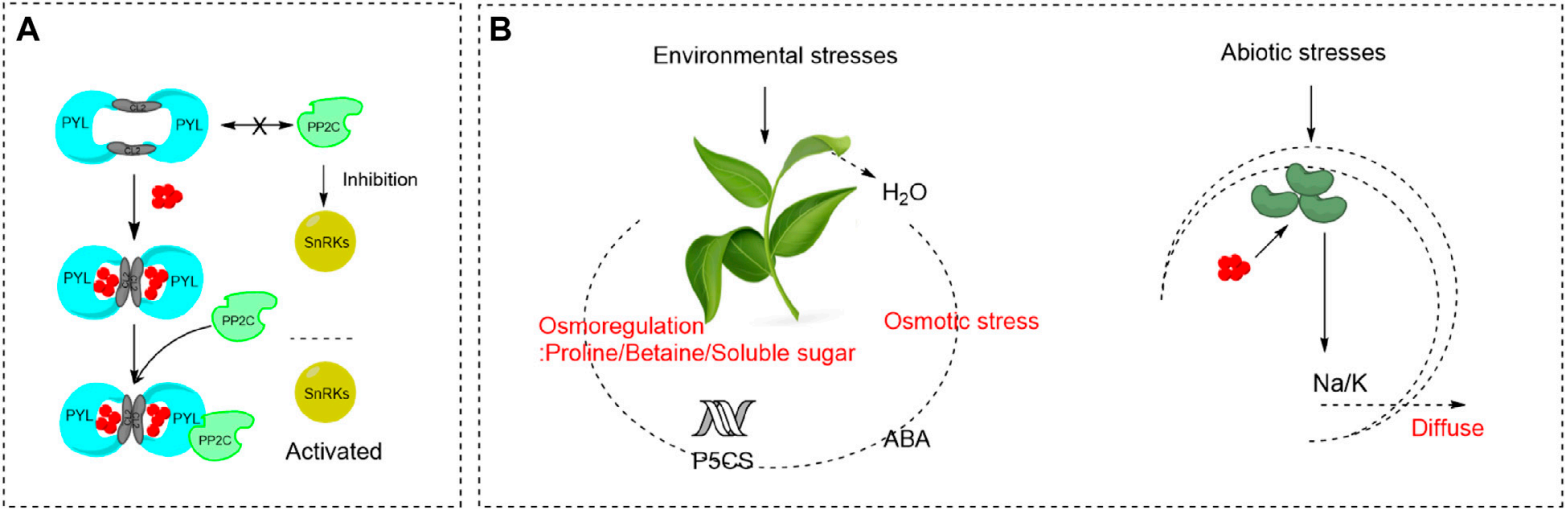
ABA signal transduction and the role of ABA pathways in stress signaling **(A)** The working model of ABA-dependent recognition and inhibition of PP2Cs by PYLs (Cutler et al., 2010; Soon et al., 2012). **(B)** The role of abscisic acid pathways in stress signaling (Shinozaki and Yamaguchi-Shinozaki, 2000; Finkelstein et al., 2002).

As shown in Figure 2A, Protein Phosphatase 2Cs (PP2Cs) and SNF1-related protein kinase 2s (SnRKs) are major negative regulators and positive regulators of ABA signaling, respectively. PYR/PYL/RCARs show conformational change upon binding of ABA, which exposes the interaction surface allowing for favorable binding of some PP2Cs (Cutler et al., 2010). Consequently, the activities of PPC2s are inhibited, allowing activation of SnRK2s. Then SnRK2s activate downstream target proteins, including basic-domain leucine zipper (bZIP) transcription factors, anion channels and NADPH oxidases, to induce ABA responses. In the absence of ABA, PP2Cs are unable to bind to PYR/PYL/RCARs and they inactivate SnRKs by dephosphorylation (Soon et al., 2012). ABA is commonly known as the “stress hormone” that responds to variety of environmental stresses, especially for abiotic stresses (Kanchan et al., 2017). When plants encounter various abiotic stresses, such as salinity, extreme temperature, heavy metal, drought, UV-B, water stresses, endogenous ABA levels increase rapidly due to the increase of ABA biosynthesis and inhibition of ABA degradation. Then the downstream specific signaling pathways are activated and gene expression levels are modified (Kanchan et al., 2017).

As shown in Figure 2B, osmotic stress caused by environmental stresses, such as high salinity, drought and cold, results in dehydration and inhibition of water uptake in plants. ABA accumulates under osmotic stress conditions and plays an important role in the stress responses and tolerance of plants (Shinozaki and Yamaguchi-Shinozaki 2000; Finkelstein et al., 2002). ABA promotes short-term responses like stomatal closure to maintain water balance (Agurla et al., 2018). For example, under salt stress, ABA is accumulated in plant roots and transported to the leaves, which leads to the decrease of leaf expansion rate and stomatal closure. As a result, the reduced transpiration rate and increased salt transportation in the root cap alleviate the damage causing by salt stress. ABA also stimulates longer-term growth responses through regulation of stress-responsive genes, the products of which may function in dehydration tolerance. Silva-Ortega et al., reported that ABA can regulate the expression of *P5CS*, which promotes the production and accumulation of osmotic adjustment substances like proline, betaine, and soluble sugar (Silva-Ortega et al., 2008).

Excepting mediating stomatal closure and osmotic regulation, ABA also involves in the balance of ion concentration under abiotic stresses. ABA participates in the modification of ATPase in plasma membrane and vacuolar membrane to provide more driving force for Na^+^/H^+^ antiporter, which strengthens Na^+^ efflux and ion regionalization to alleviate the damage of salt and alkaline stress (Janicka-Russak and Kłobus, 2007; Khadri et al., 2007). Three major pathways involved in the heavy metal detoxification can be triggered by ABA, inhibiting the uptake, altering the translocation from root to shoot, and promoting the conjugation with chelators (Hu et al., 2020). In addition, the decrease of photosynthesis caused by salt, heavy metal, alkaline and cold stress can be effectively relieved by ABA (Saradhi et al., 2010). The addition of exogenous ABA can accelerate the recovery of plant photosystem II, restore the photosynthetic inactivation state of the core complex to normal, maintain the stability of photosynthetic organs, and enhance the photosynthetic efficiency of leaf light capture and conversion (Saradhi et al., 2010). Moreover, ABA can interact with other stress resistance elements, such as antioxidant defense system and the MAPK cascades, to help plants resist environmental stresses (Danquah et al., 2014; Shu et al., 2016; Yang and Guo, 2018).

## Chemical manipulation of ABA signaling structural analogues

**FIGURE 3:**
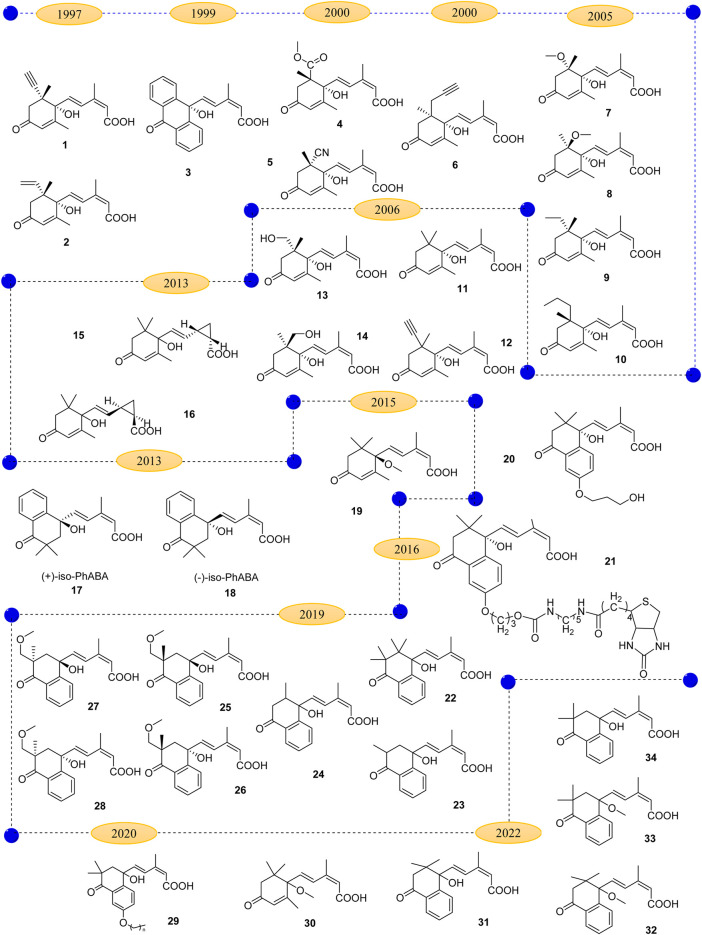
The development of abscisic acid structural analogues.

Considering the importance of ABA signaling in many processes, including stress tolerance, seed germination, root growth and development, and senescence, small molecule chemicals can manipulate ABA signaling showing potential applications in agricultural production. Among them, the most extensively studied are the analogues of ABA receptor (Bari and Jones, 2009). Up to date, many compounds have been identified as significant ABA-agonist/antagonist and can be employed to reverse the excessive/moderate ABA action, which is shown in Figure 3.

In 1997, Rose synthesized compounds **1** and **2** (Rose et al., 1997), and carried out a series of anti-stress experiments, officially started the research on the application of ABA analogues in plants. They found that both compounds **1** and **2** could stimulate the radial conduction of water in maize roots just like ABA. In subsequent studies, analogues **1** and **2** were found to reduce chloride ion concentration and ethylene content in plants. More importantly, they can delay the consumption of CO_2_ assimilation under adverse conditions. The activity of compound **2** was better than that of abscisic acid, as the activity of compound **1** was similar to that of ABA. Oxidative hydroxylation of 8′- and 9′-methyl is the key to the metabolic inactivation of abscisic acid in plants. Therefore, modification of 8′- and 9′- methyl groups to obtain more stable ABA analogues has become the research direction of many scientists.

Nicholas M. Irvine et al. synthesized an anthracenone analogue of ABA (**3**) (Irvine et al., 2000), and Takahiro Inoue synthesized new ABA analogues (**4** and **5**) (Inoue and Oritani, 2000) by regioselective hydrocyanation and possessed a cyano or methoxycarbonyl group at the 6′α-position. Bioactivity tests were performed separately, and the results show that all the compounds were less than ABA, but raised the possibility of structural modification at the 6′α-position. In the same year, Cutler found that compounds (+)-8′-acetylen-ABA (**1**) and (+)-9' -propargyl-ABA (**6**) (Cutler et al., 2000) showed better activity than ABA in inhibiting arabidopsis seed germination. Among them (+)-8′-acetylene-ABA (**1**) can inhibit not only arabidopsis seed germination, but also maize cell growth and wheat embryo germination.

Ueno found that compounds **7** and **8** (Ueno et al., 2005) had no inhibitory effect on cytochrome P450 monooxidase, which might be caused by the fact that the C-8 group was too large to bind with 8' -hydroxylase. When C-8′ methyl hydrogen is replaced by methyl and ethyl groups, 8' -methyl abscisic acid (**9**) and 8' -ethyl abscisic acid (**10**) were obtained (Ueno et al., 2005). The results showed that compound **9** was a competitive inhibitor of 8' -hydroxylase, while compound **10** had no inhibitory activity. This may be due to the fact that methoxyl and ethyl have similar stereoscopic sizes and are similarly unable to bind to the active site, so it is thought that methyl group may be the largest group tolerated by the active binding site.

Nyangulu and co-workers (Nyangulu et al., 2006) synthesized tetrahydronaphthone ABA analogue **11** by substituting benzene ring of aromatic hydrocarbon for planar propylene group structure on six rings. When the concentration was 0.33 μmol/L, the inhibition rate of ABA was about 40%, while the inhibition rate of compound **11** was less than 10%. The R configuration of compound **11** was similar to that of S configuration. Compound **12** showed a highly activity in inhibiting germination of *Arabidopsis thaliana* seeds (Nyangulu et al., 2006). Compounds **13** and **14** were synthesized on the basis of improved compounds **1** and **2** (Nyangulu et al., 2006). They suggested that when the 8' -hydroxylase oxidizes **11** to **13**, it inhibited further cyclization of compound **13** to PA analogue due to instability. Suspension culture of maize cells showed that the metabolic rate of compound **11** was similar to ABA. Compounds **13** and **14** exhibited similar activity to ABA at high concentrations. The biggest defect of ABA in practical application is poor photostability.

In order to obtain more stable ABA analogues, Liu has synthesized a photostable *cis*-2, 3-cyclopropanated abscisic acid analogue. They described the effect of ABA analogue isomers on activity. The photoisomerization study showed that compounds **15** and **16** (Liu et al., 2013) reached *cis* and inverse equilibrium in 44 and 36 h, respectively under 254 nm UV light, which was much longer than ABA (12 h) and thus had better photostability. The biological activities of compounds **15** and **16** under two modes of seed germination and seedling growth were tested. The results showed that at high concentrations (3 μmol/L and 5 μmol/L), both compounds had better inhibitory effects on seed germination than ABA did. But its inhibitory effect was lower than ABA at low concentration. The inhibitory effect of the two target compounds on the growth of *Arabidopsis thaliana* seedlings was lower than that of ABA at each concentration. Overall, compound **15** showed higher activity than compound **16**. However, in most cases, the biological activity of the *cis* structure is lower than that of the trans structure, and the difference of the results provides an important basis for further study of the structure-activity relationship of ABA.

Han et al. developed a new ABA analogue named (+) 2′,3′-*iso*-PhABA (**17** and **18**) in order to overcome its oxidative inactivation. *In vivo* experiments, the activity of (+) - *iso* -PhABA (**17**) was significantly higher than that of (+)-ABA, including inhibition of lettuce and *Arabidopsis* seed germination (the inhibititory activity on arabidopsis thaliana seed germination is about 14 times of (+)-ABA), wheat embryo germination and rice seedling elongation. Protein phosphatase 2C (PP2C) activity showed that (+) - *iso* - PhABA (**23**) was an effective and selective ABA receptor agonist, which could better bind to PYL10 (Tan et al., 2013).

With the discovery of ABA receptor proteins and the application of chemical genetics in agriculture, Chantel L. Benson synthesized a series of ABA analogues as chemical probes to understand functional redundancy in the ABA receptor family (Benson et al., 2015). In PBI 352 (**19**), the substitution of hydrogen on C-10 by methyl group for OH induced stomatal closure, but did not affect the selectivity of analogues in *Arabidopsis* seedling germination or root growth. This selectivity has potential utility in practical applications where temporary drought protection can be provided to plants without reducing concomitant root growth. For all tested RCARs, the analogue PBI 352 (**19**) significantly reduced its receptor-binding activity compared with (*S*)-ABA (**1**), and PBI 352 (**19**) binds RCAR8 to inhibit ABI2.

Rajagopalan et al., developed a library of ABA analogues containing 240 compounds that are structural variants of the basic molecule *S*-(+)-ABA. These molecules were synthesized primarily for specific structural activity studies, and one antagonist PBI686 (**20**), was identified to be active on both RCAR receptors (Rajagopalan et al., 2016). Analogues PBI686 (**20**) and PBI664 (**21**) have no agonist activity by themselves, but are effective antagonists of ABA receptors blocking phosphatase binding to receptors.

In 2019, Wan et al., designed and synthesized five analogues of an earlier 2′,3′-*iso*-PhABA (Wan et al., 2019). Bioassay results showed that the number and position of methyl groups and the substitution of hydrogen atoms on methyl groups have great influence on the activity. Compared with 2′,3′-*iso*-PhABA, dimethyl-PhABA (**22**) showed slightly decreased inhibitory activity on seed germination and seedling growth of rice. However, it significantly reduced the ability to inhibit wheat embryo germination. Both demethyl-*iso*-PhABA (**23**) and demethyl- PhABA (**24**) showed weak inhibitory activity, and 11′-methoxy- PhABA (**25**/**26**) were more potent than their isomers **30/31** in all bioassays. These results suggested that the preservation of quaternary carbon at the 2′ or 3′ position is necessary for the maintenance of ABA-like biological activity, and the demethylation at the 3′ position is more significant. Among them, **25**/**26** showed good inhibitory activity and different binding selectivity to ABA receptor PYR1 and PYL2/PYL5.

Che et al., described a class of ABA agonist/antagonist probes, APAn (**29**) that regulate either agonist or antagonist activity according to the length of the 6′-alkoxy chain (Che et al., 2020). With the extension of alkoxy chain, the receptor binding potential increased gradually, and the inhibitory activity of HAB1 decreased. Theoretical analysis based on molecular docking and molecular dynamics simulations showed that some factors outside the ligand binding pocket in the receptor also affected ligand binding to the receptor. Van der Waals interaction between alkyl chains in APAn made the ligand binding to the ABA receptor tighter. This enhanced binding makes it an antagonist rather than a weakened agonist.

Recently, Wan et al., synthesized a series of ABA analogues and tested their activities (Wan et al., 2022). The results showed that the hydroxyl group of 1′-OH is an important functional group for seed germination and development. Therefore, compound **31** and **33** can inhibit seed germination, but which is not related to stomatal movement and response to drought stress regulated by ABA and its analogues. Methylation of 1′-OH significantly reduced their ability to bind to single receptor PYL5 and slightly enhanced their ability to bind to dimer receptor PYL2. The decreased inhibitory activities of **30**, **32**, and **34** on seed germination may be related to the decreased affinity of PYL5, which is caused by the destruction of hydrogen bond network. This suggests that PYL5 is a selective receptor closely related to plant growth.

**FIGURE 4:**
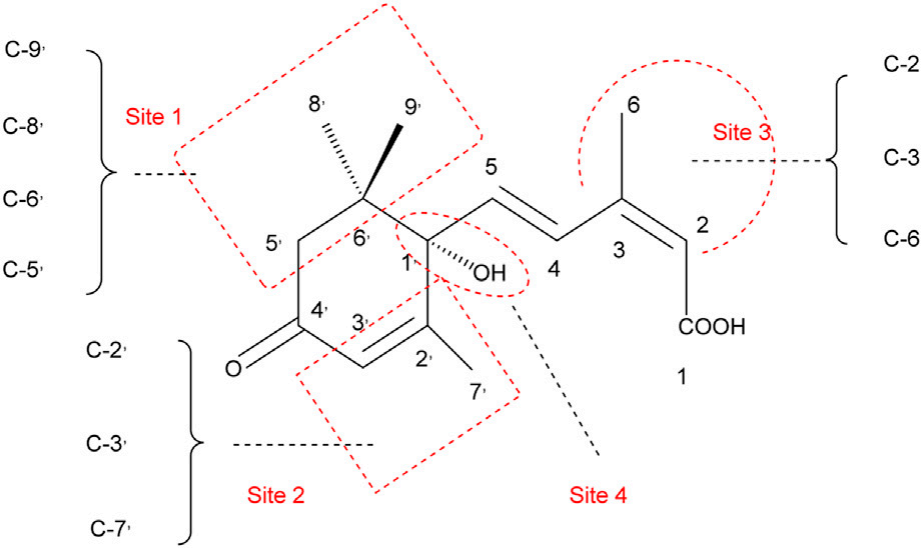
Modification sites of abscisic acid structural analogues.

The above section summarized **34** representative ABA analogues, and some have regulatory activity and some have inhibitory activity of P450 oxidase in the decomposition process, which can be used to study the molecular mechanism of ABA action and catabolism. Through the analysis of the activity or enzyme inhibitory activity of different analogues, it is known that there is a certain difference between the ABA active group and the structure inhibited by ABA 8′-hydroxylase, and this difference will help the development of new ABA analogues (An ideal enzyme inhibitor should have the ABA structural features required for substrate specificity, but not the structural features required to activate the ABA signaling pathway). Overall, the modification of site 1 (Figure 4), the spatial size and saturation will directly affect the binding ability of the compound to the target. The difference of the groups on site 2 (Figure 4) may block the binding ability of the downstream phosphatase to the receptor. The modification of site 3 (Figure 4) is to improve the high stability provides a new design idea. The modification (methylation) of site 4 (Figure 4) changes the binding ability of the analogue to the receptor by changing the hydrogen bond network.

## ABA function analogues

**FIGURE 5:**
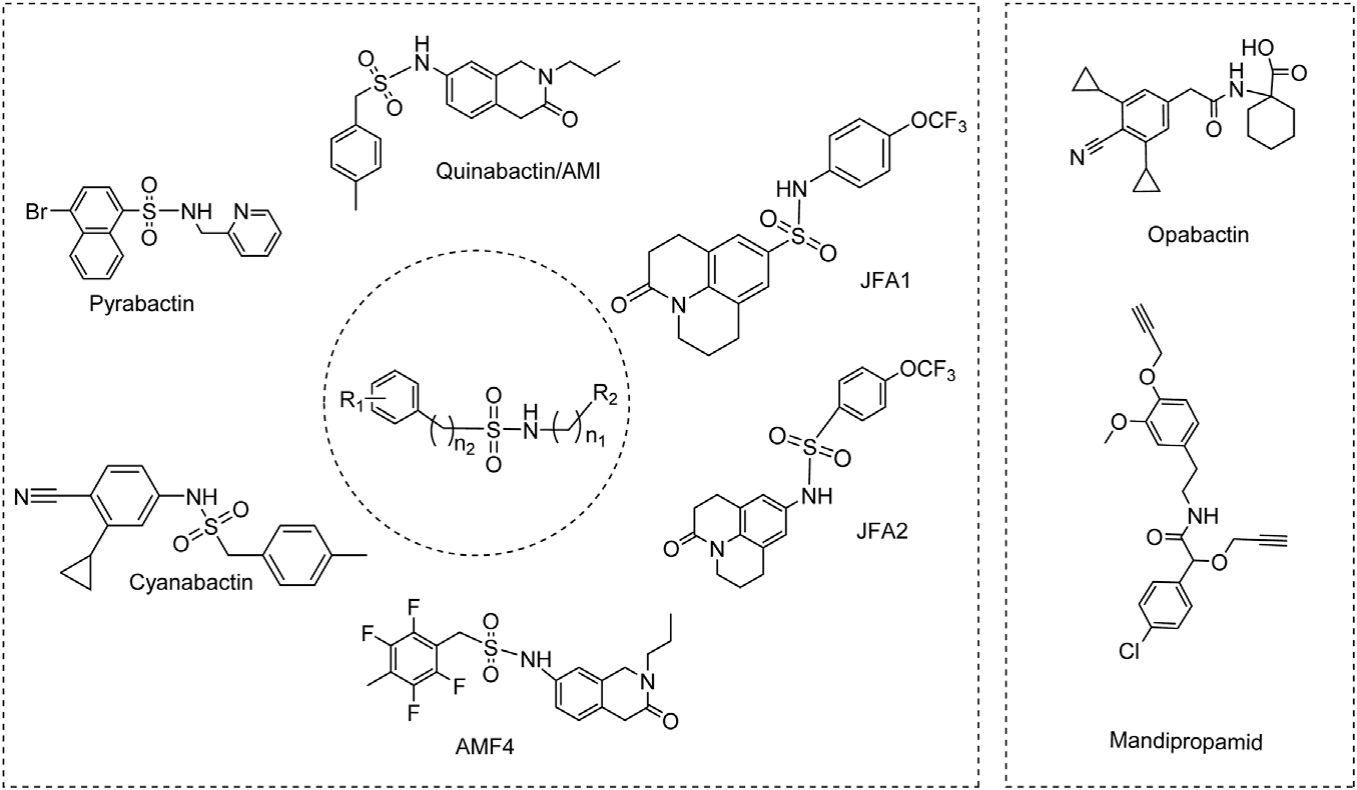
Abscisic acid functional analogues

In recent years, scientists have screened many ABA functional analogues (Figure 5) using chemical genetic methods, their structures are very different from the structure of ABA. Among which, the most representative structures are sulfonamides and amide derivatives.

Pyrabactin, a naphthalene-sulfonamide derivative, is the first synthetic ABA mimic (Park et al., 2009). Pyrabactin displayed several ABA-like activities, such as inhibition of seed germination (Park et al., 2009), induced stomatal closure (Puli and Raghavendra, 2012) and enhanced root hydraulic conductivity (Fan et al., 2015). It could be used to improve the drought tolerance of crop plants. Several structural analogues of pyrabactin have been screened and the structure-activity relationship revealed that pyridyl nitrogen is important for binding affinity biological activity (Helander et al., 2016). Studies also discovered that pyrabactin exhibits distinct responses towards PYL-subtypes. It strongly activates PYR1/PYL1 but acts as an antagonist for PYL2 and PYL3 (Melcher et al., 2010; Miyakawa and Tanokura, 2011).

Okamoto et al., identified sulfonamide AM1 (also named as quinabactin), through a yeast two-hybrid screen (Okamoto et al., 2013). Quinabactin showed a broader spectrum of PYL activity with enhanced bioactivity as compared to pyrabactin. Quinabactin treatments inhibited seed germination, prevented leaf water loss, and promoted drought resistance (Cao et al., 2013). Cao et al. developed a series of AM1 fluorine derivatives (AMFs) by progressively substituting fluorine atoms on the benzyl ring. The fluorine atoms in AMFs increase the plasma membrane permeability and hydrogen bonding network. Among these derivatives, AMF4 was proved to be a better agonist than AM1 or ABA (Cao et al., 2017). Overtveldt et al. designed phosphonamide analogues of pyrabactin, which given a different physiological response by selectively stimulating ABA-signaling pathways (Van Overtveldt et al., 2015). Further efforts exploited the structure of cyanabactin, which has a nitrile and isopropyl substituted monocyclic ring system instead of the bicyclic ring system in pyrabactin and quinabactin (Park et al., 2009; Cao et al., 2013). Using a wheat cell free-based drug screening approach, Nemoto et al. identified two novel ABA receptor agonists with bioactivity, in which JFA1 and JFA2. JFA2 was demonstrated to induce drought tolerance without affecting root growth (Nemoto et al., 2018). Cyanabactin has superior ABA-like activity in vegetative tissues and is highly effective to control plant water use (Vaidya et al., 2017). Recently, Vaidya et al. developed opabactin by a combination of virtual screening, x-ray crystallography, and structure-guided design. Opabactin is the most potent ABA agonist developed, which showed approximately sevenfold increase in receptor affinity relative to ABA and up to 10-fold greater activity *in vivo* (Vaidya et al., 2019).

As a known fungicide, mandipropamid (Figure 5) was reported to interact with a hextuple PYR1 mutant (PYR1^MANDI^) and induced stomatal closure and inhibited seed germination in transgenic plants with PYR1^MANDI^ (Park et al., 2015).

## Conclusion and outlooks

In this brief review, the research progress of ABA analogues including mechanism of action, signaling pathways, and ABA functional analogs is reviewed. the representative 34 structural analogs and 8 functional analogs are summarized, and the differences in biological activity and catabolism of ABA analogues are systematically analyzed. we conclude that the C-5^,^, C-6^,^, C-8^,^, C-9^,^ space size (functional group size) and saturations could directly affect the binding ability of the compound to the target, methyl may be the largest group that can be accepted. Differences in groups on C-2^,^, C-3^,^, C-7^,^ may block the ability of downstream phosphatases to bind to receptors. The improved photostability of C-2, C-3, C-6 provides a new design idea (cyclopropyl). Methylation of C-1^,^ alters the binding capacity of the analogues to the receptor by altering the hydrogen bonding network. Activity analysis of sulfonamide-based ABA functional analogues revealed that pyrabactin exhibits distinct responses towards PYL-subtypes. It strongly activates PYR1/PYL1 but acts as an antagonist for PYL2 and PYL3, and AM1 showed a broader spectrum of PYL activity with higher bioactivity compared to pyrabactin. Opabactin, the most potential ABA agonist to date, showed an approximately sevenfold increase in receptor affinity relative to ABA and up to 10-fold greater activity *in vivo*.

Currently, studies have revealed that PYLs can be divided into dimer receptors (PYR1, PYL1 to PYL3) and monomeric receptors (PYL4 to PYL13). However, the specific functional differences between them in the ABA signaling pathway are unclear. Utilizing chemical genetics to clarify these differences is an important means for understanding the function of PYLs proteins and an important measure to discover novel ABA functional analogues. Furthermore, on the basis of the predecessors, the in-depth research on the structure-activity relationship of ABA analogues are expected to discover ABA analogues with novel structure, higher activity and better selectivity. Foreseeably, novel ABA functional analogues will play an important role in agriculture.
